# Global Gene Expression Profiling in Three Tumor Cell Lines Subjected to Experimental Cycling and Chronic Hypoxia

**DOI:** 10.1371/journal.pone.0105104

**Published:** 2014-08-14

**Authors:** Magdalena Olbryt, Anna Habryka, Sebastian Student, Michał Jarząb, Tomasz Tyszkiewicz, Katarzyna Marta Lisowska

**Affiliations:** 1 Center for Translational Research and Molecular Biology of Cancer, Maria Skłodowska-Curie Memorial Cancer Center and Institute of Oncology, Gliwice Branch, Gliwice, Poland; 2 Institute of Automatic Control, Silesian University of Technology, Gliwice, Poland; 3 III Department of Radiation Therapy and Chemotherapy, Maria Skłodowska-Curie Memorial Cancer Center and Institute of Oncology, Gliwice Branch, Gliwice, Poland; 4 Nuclear Medicine and Endocrine Oncology Department, Maria Skłodowska-Curie Memorial Cancer Center and Institute of Oncology, Gliwice Branch, Gliwice, Poland; University Health Network, Canada

## Abstract

Hypoxia is one of the most important features of the tumor microenvironment, exerting an adverse effect on tumor aggressiveness and patient prognosis. Two types of hypoxia may occur within the tumor mass, chronic (prolonged) and cycling (transient, intermittent) hypoxia. Cycling hypoxia has been shown to induce aggressive tumor cell phenotype and radioresistance more significantly than chronic hypoxia, though little is known about the molecular mechanisms underlying this phenomenon. The aim of this study was to delineate the molecular response to both types of hypoxia induced experimentally in tumor cells, with a focus on cycling hypoxia. We analyzed *in* *vitro* gene expression profile in three human cancer cell lines (melanoma, ovarian cancer, and prostate cancer) exposed to experimental chronic or transient hypoxia conditions. As expected, the cell-type specific variability in response to hypoxia was significant. However, the expression of 240 probe sets was altered in all 3 cell lines. We found that gene expression profiles induced by both types of hypoxia were qualitatively similar and strongly depend on the cell type. Cycling hypoxia altered the expression of fewer genes than chronic hypoxia (6,132 *vs.* 8,635 probe sets, FDR adjusted p<0.05), and with lower fold changes. However, the expression of some of these genes was significantly more affected by cycling hypoxia than by prolonged hypoxia, such as *IL8, PLAU,* and epidermal growth factor (EGF) pathway-related genes (*AREG, HBEGF,* and *EPHA2*). These transcripts were, in most cases, validated by quantitative reverse transcription polymerase chain reaction (qRT-PCR). Our results indicate that experimental cycling hypoxia exerts similar, although less intense effects, on the examined cancer cell lines than its chronic counterpart. Nonetheless, we identified genes and molecular pathways that seem to be preferentially regulated by cyclic hypoxia.

## Introduction

In the tumor microenvironment, hypoxia is one of the crucial factors, which promote an aggressive phenotype of tumor cells and decrease the effectiveness of standard treatment. There are two types of hypoxia: chronic (uninterrupted) hypoxia, which is associated with an increasing distance of proliferating cells to the vessels, and cycling (acute, interrupted) hypoxia, which is mainly caused by fluctuations in the blood flow rate [1].

The existence of acutely hypoxic cells in tumors was first observed several decades ago [2] and was attributed to transient changes in blood perfusion [3]. These preliminary observations were subsequently confirmed in spontaneous animal tumors [4], experimental tumors [5], [6], [7], and in naturally occurring human tumors [8]. Recently, with the usage of pO_2_-tissue assessment technologies, the existence of cycling hypoxia was directly observed in human tumors [9]. It was estimated that tumor areas exposed to cycling hypoxia can range from 12 to 43%, (20% [3]; 35% [7]; 12% and 43% [10]), and can even be greater than the areas of chronic hypoxia in some tumors [10].

The presence of cycling hypoxia in tumors has direct consequences on the tumor behavior. Cycling hypoxia promotes spontaneous metastasis [11], [12] and the cells exposed to such conditions have even greater metastatic potential than cells exposed to chronic hypoxia [10], [13]. Cycling hypoxia also affects the effectiveness of anticancer therapies, most predominantly radiotherapy. Glioma cells grown both *in* *vitro* and as tumor xenografts, preconditioned with application of cycling hypoxia, are more radioresistant [14]. Martinive *et al.* (2006) [15] observed a similar effect for melanoma B16-F10, fibrosarcoma, and hepatocellular cancer cells cultured *in* *vitro*. Moreover, it seems that not only transient acute hypoxia affects the behavior of the cells constituting the tumor microenvironment, but also prolonged cycling hypoxia may lead to a selection of cells resistant to apoptosis and standard treatment modalities such as radiotherapy and chemotherapy [16]. This effect may be further potentiated by increased genetic instability, which has also been attributed to the hypoxic tumor microenvironment [17]. Intermittent blood flow in tumors also decreases the effectiveness of chemotherapy by limiting the delivery of drugs to tumor cells [18]. Recent studies have shown that cycling hypoxia may also be a factor in selecting and promoting cells with stem cell-like phenotype, presenting increased tumor-initiating capabilities and metastatic potential [19].

Collectively, these data suggest that cycling hypoxia, within the tumor mass, may not only cause resistance to conventional therapies, but may also facilitate a more aggressive phenotype of tumor cells. These suggestions have been supported by the clinical observation that higher degree of tumor reoxygenation after radiotherapy is associated with worse patient prognosis [20]. There are also reports showing that individual tumors differ depending on the extent of cycling hypoxia regions. Tumors that present interchangeable states of hypoxia and reoxygenation, present an increased metastatic potential [10], [12] and are more radioresistant [14]. Identification of these tumors would inevitably improve cancer prognosis and enable treatment of patients with therapy tailored to each individual case. Thus, identifying the molecular pathways and genes involved in promoting the aggressive phenotype of tumor cells under cycling hypoxia conditions seems crucial.

Herein, we investigated the influence of cycling and chronic hypoxia on gene expression profile in three cancer cell lines, using a microarray platform. The analysis indicated that cycling hypoxia exerts a similar, although weaker, influence on gene expression in cancer cells than chronic hypoxia. The main differences observed between the two types of hypoxia involved the expression of several genes such as *IL-8, CXCL2, EPHA2, AREG*, *HBEGF*, and *PLAU,* which are relevant to tumor progression. Our results indirectly suggest that cycling hypoxia may promote an aggressive phenotype by inducing the expression of genes regulating the immune response, invasion, and proliferation.

## Materials and Methods

### Cell culture and experimental design

The cell lines were purchased from American Type Culture Collection (ATCC; Manassas, Virginia, USA) and the early passages of cell cultures were used for the experiments. The cells were grown in glass plates to eliminate oxygen permeation. PC-3 prostate cancer cells and SK-OV-3 ovarian adenocarcinoma cells were cultured in RPMI medium and McCoy’s medium, respectively, supplemented with 10% fetal bovine serum (Gibco BRL, Grand Island, NY, USA). Melanoma cells (WM793B) were grown in 2% Tu medium (4:1 mixture of MCDB 153 medium with 1.5 g/l of sodium bicarbonate and Leibovitz’s L-15 medium with 2 mmol/l of L-glutamine; Sigma-Aldrich, St Louis, MO), supplemented with 2% fetal bovine serum (Gibco BRL), bovine insulin (0.005 mg/ml) and 1.68 mmol/l CaCl_2_ (Sigma-Aldrich).

**Figure 1:**
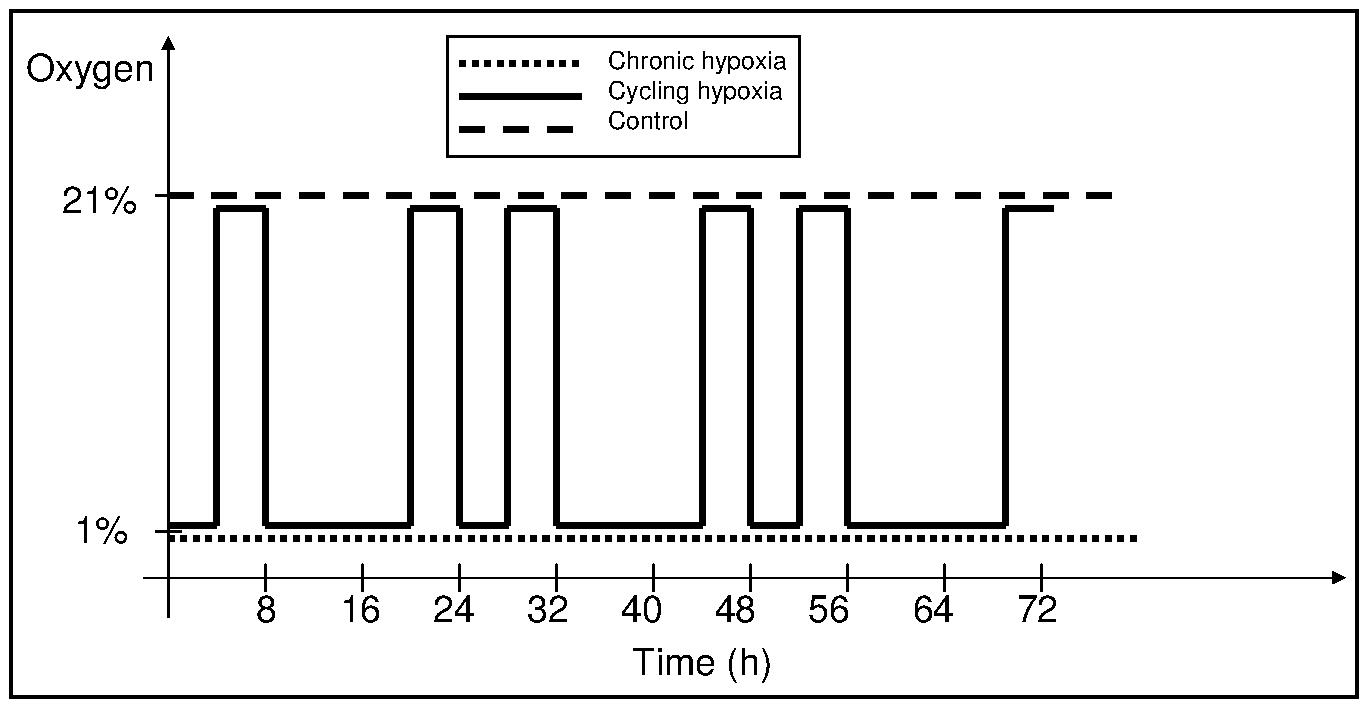
Experimental design. Cells were subjected to cycling (interchanging periods of 1% and ambient oxygen), chronic hypoxia (constant 1% oxygen) or control conditions (ambient oxygen) for 72 hours. The scheme predominantly shows the time scale of cycling hypoxia, which consisted of the following periods of hypoxia (1% oxygen) and reoxygenation (21% oxygen): 1%–4 hours, 21%–4 hours, followed by two cycles of 1%–12 hours, 21%–4 hours, 1%–4 hours, 21%–4 hours, followed by 4 hours of reoxygenation.

The cells were grown to the confluence of approx. 50–70% at 37°C in a standard humidified 5% CO_2_ incubator. Then, the control cells were left in the same conditions, while the cells subjected to chronic hypoxia experiment were transferred to the atmosphere of 5% CO_2_, 1% O_2_, 94% N_2_ for 72 hours. Cycling hypoxia was mimicked by 3 cycles of interchangeable states of hypoxia (1% O_2_) and reoxygenation (21% O_2_) following the order: 1% O_2_–4 h, 21% O_2_–4 h, 1% O_2_–12 h, 21% O_2_–4 h. Each experimental point was performed in triplicate. The experimental design is illustrated in Figure 1.

### Immunocytochemical evaluation of hypoxic condition in cell cultures

Hypoxic conditions in cell cultures were identified using the HypoxyprobeTM-1 kit (Chemicon, California, USA). Melanoma cells WM793B were grown in chamber glass slides. After two hours of incubation in 1% oxygen or in an ambient atmosphere.

pimonidazole (HypoxyprobeTM-1,) was added to the medium (final concentration 60 µg/ml). After 50 minutes of incubation the cells were fixed with 4% paraformaldehyde (pH, 7.4), stained with HypoxyprobeTM-1 antibody and DAPI. The degree of hypoxia in tumor-derived material was judged based on the 3,30-diaminobenzidine staining intensity and representative fields were photographed using a Nikon ECLIPSE E800 microscope with a Nikon FDX-35 camera (Nikon Instruments Inc., Melville, New York, USA).

### RNA isolation

Total RNA was isolated using the RNeasy Mini Kit (Qiagen, Hilden, Germany) supplemented with DNaseI (Qiagen) digestion step, according to the manufacturer’s protocol. The quantity of isolated RNA was estimated spectrophotometrically by measuring absorbance at 260 nm and the quality was assessed using 2100 Bioanalyzer (Agilent Technologies, Palo Alto, California, USA).

### cRNA synthesis and hybridization to microarrays

All procedures were performed according to the manufacturer’s instructions (Affymetrix, Santa Clara, CA, USA) using reagents recommended by Affymetrix. Total RNA (5 µg) from each sample was used as a template for cDNA and subsequent cRNA syntheses. Fragmented cRNA was hybridized to human oligonucleotide GeneChip Human Genome U133 Plus 2.0 Array (Affymetrix). Arrays were scanned by GeneChip Scanner 3000 (Affymetrix).

### Microarray Data Analysis

Obtained CEL files were pre-processed using the GC-RMA (GeneChip Robust Multiarray Averaging) algorithm available in R environment as the Bioconductor gcrma package (ver. 2.34.0). Initial filtering was applied: genes showing minimal variation across the set of arrays were excluded from the analysis (only genes whose expression differed by at least 1.5 fold from the median, in at least 20% of the arrays, were retained). This preselection led to the filtering out of more than 35 000 probe sets, thus the number of probe sets used for analysis was reduced from 54 675 ones present on each array, to the final number of 19,793 probe sets (approx. 40%). Unsupervised analysis was carried out by multidimensional scaling and visualized on a 2-D plot.

The transcripts differentially expressed between the two classes of the samples were identified by t-test using the Random Variance Model (RVM) [21]. Transcripts were considered to have a statistically significant difference in expression if the corrected p-value was less than 0.05 after Benjamini-Hochberg false discovery rate (FDR) multiple test correction [22]. Differences were deemed biologically significant if the fold change was equal or above 2. For between lists comparisons we used Venn diagrams. In certain analyses, the RVM t-test was applied individually for each cell line. When more than two groups were compared, F-test results were provided. Also, the 2-way analysis of variance (MANOVA, FDR adjusted p<0.05) was used to estimate the effect of both the hypoxia factor and the type of the cell line.

To identify the biological functions of gene groups selected in the consecutive comparisons the gene set enrichment analysis (GSEA) with c2: curated gene set collections from Molecular Signatures Database (MSigDB) or selected gene sets (289), was performed [23]. In detail, we applied two independent tests: the KS permutation test and the Efron-Tibshirani Gene Set Analysis test (GSA). We considered a GSEA category significantly differentially regulated if significance level in either of the tests was less than 0.05 after Benjamini-Hochberg false discovery rate (FDR) multiple test correction. The union of lists was used instead of intersection, as these tests assess significantly different aspects of group expression changes (GSA test estimates the global change of all genes within the list, while the KS test assesses only the change in the subgroup of samples). Analyses were performed using R (ver. 3.0.2) statistical environment with the Bioconductor software (ver. 2.13) and BRB-ArrayTools (developed by Dr. Richard Simon and the BRB-ArrayTools Development Team; ver. 4.4.0). The CELL files are deposited in NCBIs Gene Expression Omnibus (GEO, http://www.ncbi.nlm.nih.gov/geo/; Accession ID: GSE53012).

### Quantitative Reverse Transcription Polymerase Chain Reaction (qRT-PCR)

An aliquot of 1 µg or 0.5 µg of total RNA was taken for cDNA synthesis using Omniscript RT Kit (Qiagen) and random primers (4 µM, Sigma-Aldrich), oligo (dT) primer (1 µM, QBiogene Inc., Illkirch, Cedex-France) and RNase inhibitor (10 U, Fermentas, St. Leon-Rot, Germany). The reaction was performed in 20 µl total volume, according to manufacturer’s protocol, using termocycler UNO II (Biometra, Göttingen, Germany). The cDNA was diluted 10-fold and a 5-µl aliquot was taken for real-time polymerase chain reaction performed using Taqman 2x PCR Master Mix (Roche, Basel, Switzerland), Exiqon probe (100 nM, Roche) and appropriate primers (200 nM each). The reaction was carried out using ABI PRISM7700 Sequence Detection System (Applied Biosystems, Foster City, California, USA) and the following PCR conditions: 2 min at 50°C, 10 min at 95°C, 40 cycles of 15 sec at 95°C, 1 min at 60°C, and 1 min at 72°C. The expression was calculated using the modified Pfaffl model [24], (Q = E^ΔCt^, where E is reaction efficiency and ΔCt = Ct_calibrator_–Ct_sample_). The calibrator sample was a mixture of total RNA isolated from each sample. The gene expression was normalized to the expression of two genes selected by GeNorm program (ver. 3.5). These genes were as follows: for PC-3 cells: *HADHA* and *EIF5*; SK-OV-3: *HADHA* and *CTBP1*, WM793B: *CTBP1* and *EIF5*. The statistical significance of the results was estimated using Mann-Whitney U test (Statistica ver. 10). The sequence of the primers and the probe numbers are shown in Table S1.

## Results

### The hypoxia response is cell line specific

There are two major types of oxygen kinetics detected in tumor mass, depending on time scales of oxygen fluctuations, the rapid oxygen kinetic (fluctuations within seconds and minutes) and the slower oxygen kinetic (fluctuations within hours and days). While the first one is relatively well-known, the latter is not precisely characterized [25]. Here, we present a gene expression analysis in three cancer cell lines exposed to either chronic or cycling experimental hypoxia, or grown in control conditions. The cell lines used in this study represent three types of cancer, known to be affected by hypoxia (melanoma, ovarian, and prostate cancer). The cycling conditions were chosen to mimic oxygen fluctuations in a slower time scale. *In vivo,* they occur during the remodeling of the vascular network when the hypoxic and reoxygenation periods last for hours or days [25]–[27] (for details, see Materials and Methods and the experimental setup in Figure 1). To make sure that cells are exposed to hypoxia in this relatively short period of time, we evaluated the cell oxygenation state using a hypoxia marker, pimonidazole (Figure S1).

**Figure 2:**
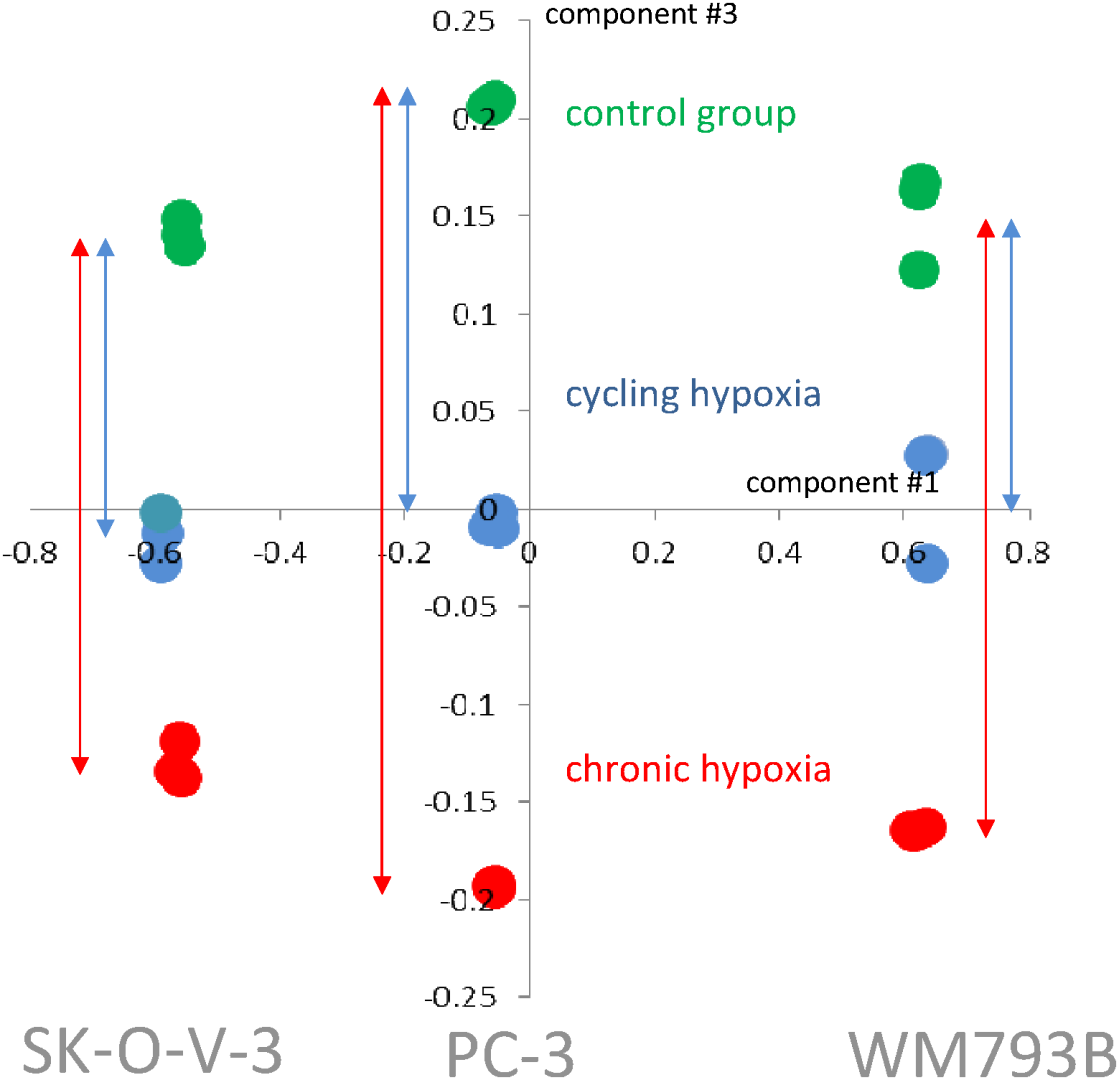
Unsupervised analysis of all samples by multidimensional scaling. The scheme shows that the difference between molecular profiles of the analyzed cell lines is the main source of variance. The hypoxic stimulus is the second factor differentiating samples. The dots represent replicas of samples: control (green), chronic hypoxia-treated (red) and cycling hypoxia-treated (blue). The axes are titled: component #1 (the X-axis) and component #3 (the Y-axis).

The experiment was performed in triplicate (27 microarrays in total). First, we performed an unsupervised analysis with a multidimensional scaling method to delineate the main sources of variability in the whole group of samples. The analysis revealed that the specific molecular profile characteristic to each cell line was the strongest factor differentiating the samples (the first and the second component), while the second factor (the third component) was the culture conditions, hypoxic *vs.* control culture conditions (Figure 2). This observation was subsequently confirmed by class comparison analysis, which revealed that as many as approximately 19,000 probe sets (out of 19,793 being analyzed) were differentially expressed between melanoma, ovarian, and prostate cancer cells, although with such a large scale of differences, a reliable estimation is difficult. Chronic hypoxia influenced the mRNA expression of 8,635 probe sets, while cycling hypoxia affected the mRNA expression level of 6,132 probe sets (Table S2).

**Figure 3:**
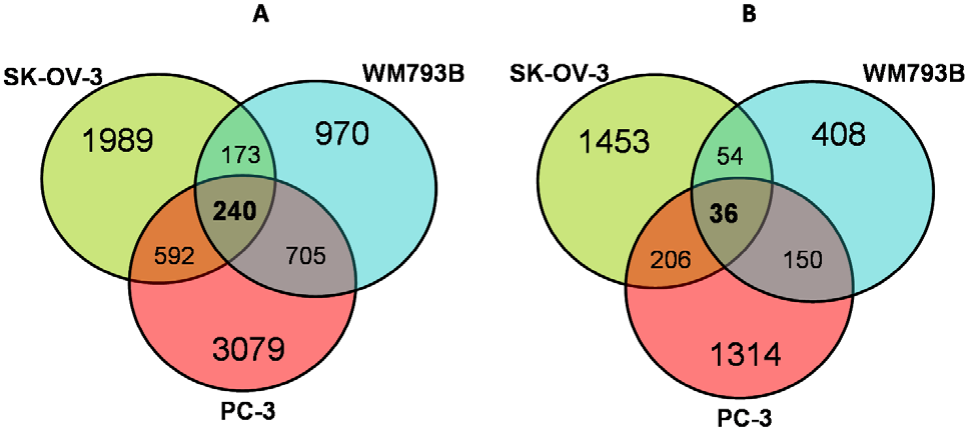
Venn diagram showing numbers of hypoxia-responsive probe-sets. The diagram shows the number of probe-sets (FDR adjusted p<0.05), for which the expression changed (FC ≥2) under either chronic (A) or cycling (B) hypoxia in comparison to control samples in each cell line, (SK-OV-3, PC-3, and WM793B) as well as the probe sets common to all or two cell lines.

Next, we analyzed how the chronic and cycling hypoxia affected gene expression in each cell line. Six comparisons were conducted for each type of hypoxia and in each cell type separately. Prostate cancer (PC-3 cell line) appeared to be the most hypoxia-responsive (4,616 probe sets), while melanoma cells (WM793B line) showed the weakest response to hypoxic conditions (2,088 probe sets). In SK-OV-3 cells, approximately 3,800 probe sets were affected by hypoxia. On the other hand, quantitatively, both ovarian and prostate cells were similarly affected by cycling hypoxia. The expression of 1,749 and 1,706 probe sets, respectively, was affected. Again, melanoma showed a relatively low response to this treatment (648 probe sets). The vast majority of selected genes were cell-line specific and only a small percentage of probe sets was overlapping between three or two cell lines. The numbers of genes (fold change (FC) ≥2, FDR adjusted p<0.05) regulated by either cycling or chronic hypoxia in each cell line are presented in the Venn diagram (Figure 3).

### Gene expression profile induced by experimental cycling hypoxia

According to the scatter plot (Figure 2), cycling hypoxia induced similar, but weaker changes in gene expression profiles when compared to those induced by chronic hypoxia. We arbitrarily defined the “cycling hypoxia-responsive genes” as those whose expression was not affected or affected to a much lesser extent, or reversely modulated by chronic hypoxia. The comparison analysis of the merged chronic hypoxia samples *vs.* the merged cycling hypoxia samples did not allow the identification of genes, which met the above criteria. However, 36 probe sets responded to cycling hypoxia in all three cell lines (fold change >2 and FDR adjusted p<0.05); presented in Table S3). Nonetheless, all of them were also significantly affected by chronic hypoxia and could not be regarded as specifically regulated by cycling hypoxia.

Thus, we decided to analyze the gene expression profile induced by cycling hypoxia in each cell line separately. We aimed at selecting genes (FDR adjusted p<0.05), which met the following arbitrarily set criteria, their expression was affected by cycling hypoxia conditions at least twice (cycling samples *vs.* controls FC ≥2) and not affected or reversely modulated, or significantly less modulated by chronic hypoxia (cycling *vs.* chronic samples, FC ≥1.5).

We found 419, 61, and 33 probe sets, which met the above criteria for SK-OV-3, PC-3, and WM793B cell lines, respectively. In ovarian cancer cells, the majority of selected genes were induced, while, in PC-3 and melanoma cells, most cycling hypoxia-regulated genes were suppressed. The gene lists differed between the cell lines, but some transcripts were overlapping, including those of *AREG*, *CXCL2,* and *PLAU*, while *HBEGF* was marginal regarding these criteria. Interestingly, *CXCL2* regulation by hypoxia depended on the cell line. *CXCL2* was induced by cycling hypoxia in ovarian cancer cells and repressed in melanoma. The top 20 cycling hypoxia-responsive genes are presented for each cell line in Table 1. The whole “cycling hypoxia-responsive gene” lists for each cell line are presented in Tables S4–S6.

**Table 1:** Cycling hypoxia-responsive genes in different cell lines (FDR adjusted p<0.05; the highest fold change between cycling vs. chronic and/or chronic vs. cycling hypoxia).

SK-OV-3				PC-3				WM793B			
Genesymbol	GeneID	Cyclingvs.control(foldchange)	Chronicvs.control(foldchange)	Genesymbol	GeneID	Cyclingvs.control(foldchange)	Chronicvs.control(foldchange)	Genesymbol	GeneID	Cyclingvs. control(foldchange)	Chronicvs. control(foldchange)
*E2F7*	228033_at	+10.2	+5.7	*DHRS9*	224009_x_at	+17.5	+7.4	*TRIM73*	1554250_s_at	+3.3	+2.0
***IL8***	211506_s_at	+9.13	+1.8	*CEACAM5*	201884_at	+9.7	+6.8	*C17orf91*	214696_at	+2.4	–1.1
*THBS1*	201109_s_at	+8.9	+1,8	*PDK3*	206348_s_at	+6.6	+4.2	*RASGRP1*	205590_at	–2.0	+1.8
*KITLG*	226534_at	+9.6	+4.0	*SERPINB2*	204614_at	+5.9	+2.0	*FAM13A*	217047_s_at	–2.0	1.0
*FAM83D*	225687_at	+6.3	+3.0	*IL1RN*	212657_s_at	+5.3	+3.0	*ARHGAP24*	223422_s_at	–2.0	1.0
*STK17A*	202695_s_at	+5.7	+2.2	*SHANK2*	243681_at	+4.4	+0.9	*BIRC3*	210538_s_at	–2.1	+1.1
***HBEGF***	38037_at	+5.7	1.0	*ST6GAL2*	228821_at	+4.05	+2.6	*TNFAIP3*	202643_s_at	–2.1	+1.4
*IRAK2*	231779_at	+5.3	+2.2	*CEACAM6*	211657_at	+3.5	+1.9	*MTSS1*	203037_s_at	–2.1	–1.3
*PHLDB2*	238419_at	+5.2	+2.4	HBEGF*	38037_at	+3.2	+2.3	*UBASH3B*	238587_at	–2.1	–1.3
*MKI67*	212020_s_at	+5.05	+2.9	*EDN1*	222802_at	+3.0	+2.0	*TNFAIP3*	202644_s_at	–2.1	1.0
***EPHA2***	203499_at	+4.4	+1.8	*AREGB*	1557285_at	+3.0	+1.4	*ABTB2*	213497_at	–2.2	–1.2
***PLAU***	211668_s_at	+4.2	+2.1	*CBLB*	227900_at	+3.0	+1.4	*IRAK2*	231779_at	–2.4	–1.3
*DUSP6*	208893_s_at	+3.3	–1.4	*AREGB*	1557285_at	+2.9	+1.4	*LINGO2*	232720_at	–2.4	–1.5
***AREG***	205239_at	+2.7	–6.5	*ARL14*	220468_at	+2.9	+1.2	*NFKBIZ*	223218_s_at	–2.7	–1.4
*IL1A*	210118_s_at	+2.7	–1.6	***PLAU***	211668_s_at	+2.4	+1.4	*CXCL3*	207850_at	–2.9	–1.2
*IL6*	205207_at	+2.5	–1.7	***AREG***	205239_at	+2.4	+1.1	*ZMYND8*	209049_s_at	–3.3	–2.1
***CXCL2***	209774_x_at	+2.3	–3.9	*ZNF846*	1569157_s_at	+2.3	1.0	*MIRHG2*	229437_at	–4.4	–2.8
*EREG*	205767_at	+2.15	-12.7	*HSPA6*	213418_at	–3.6	–1.5	*PDE4B*	203708_at	–4.5	–1.9
*IGFBP1*	1558605_at	–3.4	+1.7	*PSMB4*	228204_at	–4.7	+1.8	***CXCL2***	209774_x_at	–4.5	–1.3
*INSIG1*	201626_at	–3.9	+1.9	*RPLP2*	200908_s_at	–6.0	+1.1	*ACSL6*	213683_at	–6.8	–4.4

The genes in **bold** have been chosen for qRT-PCR validation. The gene marked with * was marginal regarding the criteria described in the text.

To find out what biological processes are regulated preferentially by cycling hypoxia, a Gene Set Enrichment Analysis (GSEA) was performed. The analysis was done for each cell type separately and the results are presented in Table S7. First, comparisons were conducted for each type of hypoxia and in each cell type (6 comparisons in total). Among the total number of about 2,900 gene sets selected (FDR corrected p<0.05 in either KS test or GSA test), the majority of genes was affected by both types of hypoxia. Since we were interested in gene sets predominantly affected by cycling hypoxia, signatures which were significantly affected in samples subjected to cycling hypoxia, but not affected in samples subjected to chronic hypoxia were identified (FDR adjusted p<0.05 in GSA and KS tests in comparison “cycling *vs.* control” and FDR adjusted p>0.05 in comparison “chronic *vs.* control”). There were 43, 85, and 40 gene sets that were preferentially affected by cycling hypoxia in prostate, ovarian, and melanoma cells respectively (marked with colors in Table S7). The vast majority of the gene sets was specific to a particular cell line. However, five signatures were common to two cell lines (Zucchi_Metastasis_dn, Brueckner_Targets_of_MIRLET7A3_dn, Reactome_Activation_of_Chaperone_Genes_by_XBP1S, Reactome_Metabolism_of_Nucleotides, Zhong_Response_to_Azacitidine_and_TSA_up). Most of the genes constituting the aforementioned signatures were different between the cell lines, except for chemokines (e.g., *CXCL2, CXCL1, CXCL3,* and *CXCL20*). Their expression was induced in both ovarian and prostate cancer cells, while repressed in melanoma cells. Some signaling pathways were predominantly affected within a particular cell line, e.g., pathways related to TP53 or NFκB in prostate cells, the epidermal growth factor (EGF), the activator protein 1 (AP1), and immune response related pathways in ovarian cancer, while fatty acids metabolism and response to exogenous chemicals associated pathway were affected in melanoma cells (marked with colors in Table S7). Since the abundance and the variability of the gene sets made it difficult to draw precise conclusions, we decided to perform a GSEA with gene sets related to selected processes (proliferation, cell cycle, replication, migration, adhesion, invasion, stress response, immune response, DNA repair, and hypoxia response) and signaling pathways (hypoxia inducible factor (HIF), NFκB, Ap1, EGF, and vascular endothelial growth factor (VEGF)). The analysis of selected gene sets (289) confirmed that transcripts linked to the NFκB, AP1, as well as VEGF and EGF signaling pathways were more affected by cycling hypoxia than chronic hypoxia in ovarian and prostate cancer cells; while those related to immune response and replication were preferentially affected in melanoma cells exposed to cycling hypoxia. In addition, gene sets related to invasiveness were slightly more represented in SK-OV-3 cells subjected to cycling hypoxia than in SK-OV-3 cells subjected to chronic hypoxia. The difference between cycling and chronic hypoxia conditions was even more significant when gene sets, which were significantly affected in either KS or GSA test, were taken into account in the analysis. In these settings, genes linked to adhesion, immune response, cell cycle, and proliferation were more affected in ovarian cancer cells exposed to cycling hypoxia than in cells subjected to chronic hypoxia, while, in prostate cancer cells, genes involved in DNA repair seemed to be more responsive to cycling hypoxia than to chronic hypoxia. For HIF and hypoxia signaling pathways, no differences were observed between cycling and chronic hypoxia treatment. The results are presented in Table S8.

### “Universal” chronic hypoxia genes

In search for genes of core response to hypoxia, 240 probe sets, which displayed a statistically significant (FDR adjusted p<0.05) change in mRNA expression under hypoxic conditions across all three cell lines (fold change ≥2) were identified. The expression of 30 genes was induced five or more times in hypoxic conditions. The chosen ones are presented in Table S9. Among the selected genes, some are well-documented as hypoxia-regulated genes (e.g., *CA9, VEGF, ADM, BNIP3, RNASE4,* and *SLC2A1*), some weakly documented (e.g., *ANKRD37, STC1, STC2,* and *TMEM45A*) and some have not been linked with hypoxia so far (e.g., *TAF9B* and *ARRDC3*). The entire gene list is presented in supplemental data (Table S10).

Again, the analysis revealed that a vast majority of genes responding to hypoxia is cell type specific. Among them, numerous genes are known to be related to carcinogenesis and/or tumor progression such as *BRAF* (the main oncogene in melanoma), *NEDD9, LOX, CXCR4* (related to cancer spreading), DNA repair genes (*BRCA1* and *BRCA2*), angiogenesis regulators (*ADM, ANG,* and *ANGPTL4*), and cancer suppressor genes (*NME1* and *WISP2*).

The arbitrarily selected genes, regarded as the most interesting biologically, are presented in Table 2. A full list of hypoxia-responsive genes selected for each particular cell line is available as supplemental data (Tables S11–S13).

**Table 2:** Hypoxia-regulated genes strongly related to carcinogenesis.

GENE	ID	Role in cancerbiology	WM793B(Foldchange)	PC-3(Fold change)	SK-OV-3(Fold change)
*ADM*	202912_at	Angiogenesis	+4.9	+20.1	+17.5
*ANG*	205141_at	Angiogenesis	+5.5	+2.7	+11.4
*ANGPTL4*	223333_s_at	Angiogenesis	+11.4	+93.7	+42.4
*ATF3*	202672_s_at	Pro- and antitumorogenic,invasiveness	+2.06 (p = 0.0022)	+7.4	–7.3
*BRAF*	206044_s_at	Main oncogene inmelanoma	+3.55 (p = 0.0055)	-	-
*BRCA1*	211851_x_at	DNA repair, stabilitygene	–4.1	-	-
*BRCA1*	204531_s_at	DNA repair, stabilitygene	–3.9 (p = 0.0036)	–3.45	-
*BRCA2*	214727_at	DNA repair, stabilitygene	–2.2	-	-
*CADM1*	209032_s_at	Tumor suppressor	-	-	+4.5
*CXCL12*	209687_at	Drug resistance,metastatic spreading	–10.9	-	-
*CXCR4*	217028_at	Drug resistance,metastatic spreading	-	+25.6	+264.1
*CXCR7*	212977_at	Drug resistance,metastatic spreading	-	+10.5	+38.15
*EREG*	205767_at	Proliferation, cellsurvival	-	-	–12.7
*FGF1*	1552721_a_at	Proliferation,resistance to celldeath, angiogenesis,invasiveness	-	+16.6	-
*FN1*	210495_x_at	Adhesion, migration,invasiveness	+2.0 (p = 0.0013)	+1.3	-
*ICAM2*	213620_s_at	Immune surveillance	–	-	+10.4
*KITLG*	226534_at	Tumor growth, anti-apoptosis	–	-	+4.06
*LOX*	215446_s_at	Metastasis,nvasiveness, EpithelialMesenchymalTransition (EMT),	+4.7	+4.4	+1.7
*MCAM*	209087_x_at	Invasiveness, tumorsuppressor	-	–2.15	-
*MCAM*	211042_x_at	Invasiveness, tumorsuppressor	–2.1	–2.3	+1.9
*NEDD9*	202149_at	Invasiveness, metastasis	-	–2.55	+5.7
*NME1*	201577_at	Metastasis suppressor	–1.6 (p = 0.0033)	–2.7	-
*OSMR*	1554008_at	Invasiveness,	+5.8	-	+7.9
*SERPINE1*	202628_s_at	Invasiveness, metastasis	-	+1.7	+10.8
*SERPINE1*	202627_s_at	Invasiveness,metastasis	-	+6.4	+17.4
*STAT1*	200887_s_at	Proliferation, cellgrowth	–1.7	–2.45 (p = 0.0013)	+2.2
*WISP2*	205792_at	Tumor suppressor inbreast and pancreaticcancer	-	-	+183.4

“+” and “–” signify increase and decrease in expression, respectively.

The core hypoxia-responsive processes were also revealed in GSEA. More than 40 signatures were significantly affected by hypoxia in all cell lines, most of which were associated with hypoxic stimulus itself or known processes regulated by hypoxia such as glycolysis or HIF-targets. There were also gene sets related to cancer signaling pathways (*MYC*, *STAT3*, and *CDH1*) or genes moderately involved in cancer (*IRF4*, *SOX4*, and *EZH2*), as well as some associated with various aspects of cancer such as cell transformation, metastasis, and response to therapeutics (Table S7).

### Validation of the results by quantitative reverse transcription- polymerase chain reaction (qRT-PCR) analysis

Out of the genes that were classified as more affected by cycling hypoxia than chronic hypoxia, six genes were chosen for verification by qRT-PCR. The selected genes had the highest fold change (FC) when comparing “cycling *vs.* control,” the highest FC between chronic and cycling hypoxia, and/or were found to be hypoxia-regulated in more than one cell line. These genes were *Il8, PLAU, EPHA2, AREG, CXCL2,* and *HBEGF.* The selected genes were validated in independent material (see Materials and Methods section). Generally, we confirmed the tendency observed in the microarray analysis for most genes and cell lines (p<0.05, Mann-Whitney U test). However, the results obtained for the SK-OV-3 cell line were only marginally significant (p = 0.051), probably due to the low number of samples (three). The genes that were not validated in qRT-PCR analysis were *EPHA2* and *PLAU* for SK-OV-3 cells and *HBEGF* for PC3 cells. On the other hand, *EPHA2* was differentially expressed in PC-3 cells subjected to cycling hypoxia, although this was not detected by microarray analysis. The results are presented in Table 3.

**Table 3:** Validation of the results by qRT-PCR analysis.

Gene	SK-OV-3cycling vs.control foldchange(array/qRT-PCR)	SK-OV-3chronic vs.control foldchange(array/qRT-PCR)	PC-3 cyclingvs. controlfold change(array/qRT-PCR)	PC-3 chronicvs. controlfold change(array/qRT-PCR)	WM793Bcycling vs.controlfoldchange(array/qRT-PCR)	WM793B chronic vs. control foldchange (array/qRT-PCR)
***Il8***	9.1**/** **6.2^#^**	1.8**/2.2^#^**	6.5/3.5	9.2/4.5	0.9/0.65	1.0/1.0
***AREG***	2.7**/1.75^#^**	0.15**/0.5^#^**	2.4/1.8*	1.15/0.8*	Low or noexpression	Low or noexpression
***HBEGF***	5.7**/2.25^#^**	1.0**/1.45^#^**	3.0/0.86	2.4/1.8	Low/1.45	Low/0.75
***EPHA2***	4.4/2.2*	1.8/2.1*	1.0/1.3*	0.82/0.8*	1.3/1.25*	0.9**/1.0**
***PLAU***	4.2/1.8	2.1/1.6	2.4/2.1*	1.4/0.6*	Low or noexpression	Low or noexpression
***CXCL2***	2.3**/2.9^#^**	0.25**/1.6**	3.4/3.1*	3.7/2.3*	0.2/0.3*	0.8**/0.7**

In bold are results showing the differential gene expression between cycling and chronic samples assessed by qRT-PCR. The results marked with * are statistically significant (p<0.05; U Mann-Whitney test), whereas those marked with # have p = 0.051.

## Discussion

### Cycling *vs*. chronic hypoxia

In this study, we present a report on global gene expression analysis in three tumor cell lines subjected to experimental cycling and chronic hypoxia. Since there is more and more data on the role of cycling hypoxia in tumor cell aggressiveness and resistance to therapies, we aimed at identifying the molecular response to those stressing conditions in tumor cells.

The unsupervised analysis of the microarray data revealed that molecular differences between the examined cell lines were the main source of variance. This observation is easily explainable since each cell line comes from a single tumor, which is a unique genetic and molecular entity [28]. Additionally, our cell lines were derived from different tissues. Hypoxia was the second factor differentiating the samples, causing a change of expression in the range of approximately 10–23% (chronic) and 3–8% (cycling) genes. For chronic hypoxia, the response was much larger than that observed previously e.g., 10.3% [29], 1.5% [30], and 0.5% [31]. The observed discrepancies may be the result of technical (data analysis methods), experimental (relatively long exposure to hypoxia), and biological (cell type) factors. The last one influenced not only the number of affected genes, but also gene expression patterns, which were cell line-specific. This observation is in line with commonly reported phenomenon of the inter-cellular heterogeneity in molecular response to hypoxia [29]. Since the molecular response to cycling hypoxia was not largely reported, it was the focus of our study.

The comparison between chronic and cycling hypoxia revealed that, generally, the expression profiles induced by these two types of hypoxia were similar, though the latter one had a weaker effect on the transcriptome, as manifested by a lower number of affected genes and a lower fold changes. In our data, the expression of known HIF1-regulated genes was less affected by cycling hypoxia than by chronic hypoxia, suggesting that HIF1 is more active during chronic hypoxia. This result contrasts with previous data, which showed that acute hypoxia activates HIF1 more strongly than chronic hypoxia [14], [15], [32]. Since the aforementioned results come from analyses of cycles of hypoxia and reoxygenation lasting for seconds or minutes, one may speculate that the strong HIF1 activation occurs preferentially in cells exposed to short-time cycles of hypoxia and reoxygenation rather than cycle of hypoxia lasting hours or days. This is supported by Weinmann *et al.* observations. Exposition of HCI H460 cells (lung carcinoma cells) to 10 cycles of hypoxia (48 h) and reoxygenation (120 h) not only did not induce HIF1-regulated genes, but also suppressed a substantial number of them [16]. Moreover, one cannot exclude that what is actually being measured is more the effect of reoxygenation rather that cycling hypoxia itself. The limitations in interpretations of the data associated with cycling versus chronic hypoxia and reoxygenation are, at least, partially caused by the lack of a defined experimental *in* *vitro* model of cycling hypoxia [33]. A systematic microarray or PCR analysis at various time points of experimental cycling hypoxia would provide additional information on this issue.

Our experimental model did not allow us to identify a gene set more significantly affected in all three cell lines by cycling hypoxia rather than by chronic hypoxia. This finding may be explained by two facts. Firstly, gene expression profiles induced by cycling hypoxia did not differ much from those induced by chronic hypoxia, and, secondly, the cell line-specificity was relatively high in cycling hypoxia-responsiveness. Thus, we decided to select transcripts separately for each cell line. The numbers of identified genes differed between the cell lines and were consistent with the intrinsic hypoxia-responsiveness of the analyzed cells, with SK-OV-3 being the most responsive and WM793B the least. A substantial number of genes selected for prostate cancer and melanoma cells presented a relatively low expression. However, the expression of most of them were above the background noise level, which, based on Gaussian mixture decomposition method [34], was estimated to be 5. qRT-PCR analysis also confirmed the calculation since the results obtained for *HMGB*, its expression ranged between 15.3 and 52.74 in SK-OV-3 cells, was validated by this method.

Among the most induced genes selected for SK-OV-3 cells, some were involved in the regulation of proliferation such as *E2F7* and some genes belonged to the EGF pathway, either ligands (*AREG, HBEGF,* and *EREG*) or its target gene (*EPHA2*). All of them encode for proteins known to play a role in cancer progression. E2F7 is involved in resistance to therapy [35], while EPHA2 is overexpressed in a substantial number of tumors and promotes metastasis by stimulating tumor cell migration, invasion, and angiogenesis [36]. The AREG protein guarantees tumor cell self-sufficiency in generating growth signals, limitless replicative potential, and resistance to apoptosis [37]. EREG prognostic value has been demonstrated [38], while HBEGF was shown to regulate tumor cell metastatic phenotype [39].

Some of the aforementioned genes (*AREG* and *HBEGF*), together with *AREGB,* were also induced by cycling hypoxia in PC-3 cells, though *AREG* was not confirmed by qRT-PCR. On the other hand, *EPHA2* was upregulated in WM793B under cycling hypoxia. The fold changes for the aforementioned genes were relatively low, though, for *HBEGF* and *EPHA2,* the results were statistically significant in qRT-PCR analysis.

The preferential regulation of the EGF pathway by cycling hypoxia was also observed in GSEA, which showed a predominance of gene sets associated with EGF in cycling samples subjected to hypoxia, especially in ovarian and prostate cancer cells. Since the EGF pathway is generally involved in establishing and maintaining the malignant phenotype, our results suggest that this pathway may contribute to cycling hypoxia-related adverse effects. GSEA also confirmed that exposition to hypoxia and reoxygenation affected the NFκB and AP1 signaling pathways, which have been previously shown to be involved in cellular response to transient/acute hypoxia [40], [41].

The other group of genes induced by cycling hypoxia in SK-OV-3 cells comprised transcripts of proinflammatory cytokines such as *IL8, IL6, and IL1A* and chemokine such as *CXCL2. CXCL2* was also slightly more induced by cycling hypoxia than by chronic hypoxia in PC-3 cells. In contrast, in melanoma cells, its expression was significantly suppressed under cycling hypoxia. All these genes are known to play a role in cancer biology. The predominance of the regulation of immune response genes by cycling hypoxia in melanoma and ovarian cancer cells was also evidenced by GSEA. Moreover, in ovarian cancer cells, sets of genes regulating adhesion, migration, and invasion as well as proliferation and cell cycle proved to be more responsive to cycling than to chronic hypoxia. However, the flow cytometry analysis did not show any differences in cell cycle either in samples subjected to chronic or cycling hypoxia compared to control samples (data not shown).

In contrast to SK-OV-3 and PC-3 cells, the melanoma cell line appeared to be the least responsive to cycling hypoxia. Gene expression was rather suppressed than stimulated in these cells. The fold changes were also relatively low. Moreover, out of the six validated genes, qRT-PCR only allowed us to confirm the differential expression of *EPHA2* and *CXCL2*. *EPHA2* has a significant role in melanoma biology as an oncogene and pro-malignant protein [42], while *CXCL2* is a neutrophil attractant. *CXCL2* down-regulation is related to inhibition of neutrophil accumulation and their survival [43]. Thus, *EPHA2* and *CXCL2* potential role in cycling hypoxia-induced melanoma progression is worth further investigation.

### “Universal” hypoxia genes

We selected 240 probe sets representing approximately 190 genes, which presented a variable, though consistent response to hypoxic conditions in all the three cell lines. The gene set contains both well-known hypoxia-responsive genes and, more interestingly, some genes that were not linked to hypoxic stimulus or genes for which hypoxia-responsiveness is not well established. The first group consists of the most common hypoxia markers: *CAIX, GLUT1,* and *VEGF* as well as glycolytic genes (*ALDOC, ENO2, HK2,* and *PFKP*), and others (*BNIP3, ADM*, *NDRG1, RNASE4,* and *MXI1*), which appear in most of the previously identified hypoxic signatures [30], [44]–[47], including those selected under severe hypoxia [29], [48]. Their elevated expression may be treated as a validation of our experimental setting. The novel, most modulated hypoxia-responsive genes detected in our study are *TAF9B* and *ARRDC3*. These genes encode proteins that, among other functions, play roles in processes that presumably help the cell to survive hypoxic and stress conditions by regulating its viability, transcription repression (*TAF9B*, [49], and energy expenditure (*ARRDC3,* [50]). Additionally, our data confirmed the involvement of certain genes, which have been modestly reported so far, in hypoxia response. These include previously identified HIF-1 target genes such as *ANKRD37* [47], *STC-2* [51], and *STC-1* [52], [48], as well as *COL5A1* induced by hypoxia in the ventricle [53], *ZNF395* regulated by hypoxia in glioblastoma [54], and *TMEM45* in hematopoietic stem cells [55]. Some of them have also been shown to be involved in cancer progression such as *STC1* [56] and *ZNF395* [57]. Most of the aforementioned genes were highly induced by hypoxia in the analyzed cell lines and, in conjunction with the known hypoxia genes, they could be used as putative universal hypoxia signatures for further analysis and validation in clinical material. Prognostic values of some hypoxic signatures based partially on *in* *vitro* data were already successfully validated in clinical material [58], [59]. On the other hand, a huge study by Starmans *et al*., (2012) suggested that hypoxia-regulated genes may constitute a part of prognostic signature rather than be significant as individual molecular prognostic markers [60].

In conclusion, our global gene expression analysis of three tumor cell lines exposed to cycling and chronic hypoxia revealed substantial similarities in the molecular profiles induced by these two different experimental hypoxic conditions. Additionally, we have selected genes and indicated processes, which seem to be preferentially regulated by cycling hypoxia and reoxygenation, but which are not regulated or weakly regulated by chronic hypoxia in selected tumor cell types. Despite the fact that the identified genes were relatively cell line-specific, induction of some EGF pathway-related transcripts in the analyzed cell lines suggests that this molecular pathway may be involved in the tumor cell response to cycling hypoxia. Conceivably, they may mediate cycling hypoxia-induced tumor aggressiveness, though, due to the lack of established models of cycling hypoxia, caution in data interpretation is recommended. Nevertheless, these genes as well as newly reported more universal hypoxia-responsive genes are worth further validation.

## Supporting Information

Figure S1 **Pimonidazole-staining of WM793B cells after 2 hour incubation in hypoxic atmosphere (1% O_2_).**
(TIF)

Table S1 **Sequence of the primers used for qRT-PCR analysis.**
(DOC)

Table S2 **Multivariate analysis.**
(DOC)

Table S3 **Cycling hypoxia-responsive genes common to all three cell lines.**
(XLS)

Table S4 **Cycling hypoxia-responsive genes selected for SK-OV-3 cell line.**
(XLS)

Table S5 **Cycling hypoxia-responsive genes selected for PC-3 cell line.**
(XLS)

Table S6 **Cycling hypoxia-responsive genes selected for WM793B cell line.**
(XLS)

Table S7 **The results of GSEA with c2: curated gene set collections MSigDB (3818 Gene Sets).**
(XLSX)

Table S8 **The results of GSEA with gene sets for selected processes and signaling pathways (289 Gene Sets).**
(XLSX)

Table S9 **Selected hypoxia-regulated genes common to all three tumor cell lines.**
(DOC)

Table S10 **The entire gene set of hypoxia-regulated probe sets common to all three cell lines.**
(XLS)

Table S11 **Hypoxia-responsive genes selected for SK-OV-3.**
(XLS)

Table S12 **Hypoxia-responsive genes selected for PC-3.**
(XLS)

Table S13 **Hypoxia-responsive genes selected for WM793B.**
(XLS)
