# Supplementary figures and images for: Global Gene Expression Profiling in Three Tumor Cell Lines Subjected to Experimental Cycling and Chronic Hypoxia
Source: PLoS One. 2014 Aug 14;9(8):e105104. doi: 10.1371/journal.pone.0105104 (PMC4133353; doi:10.1371/journal.pone.0105104)

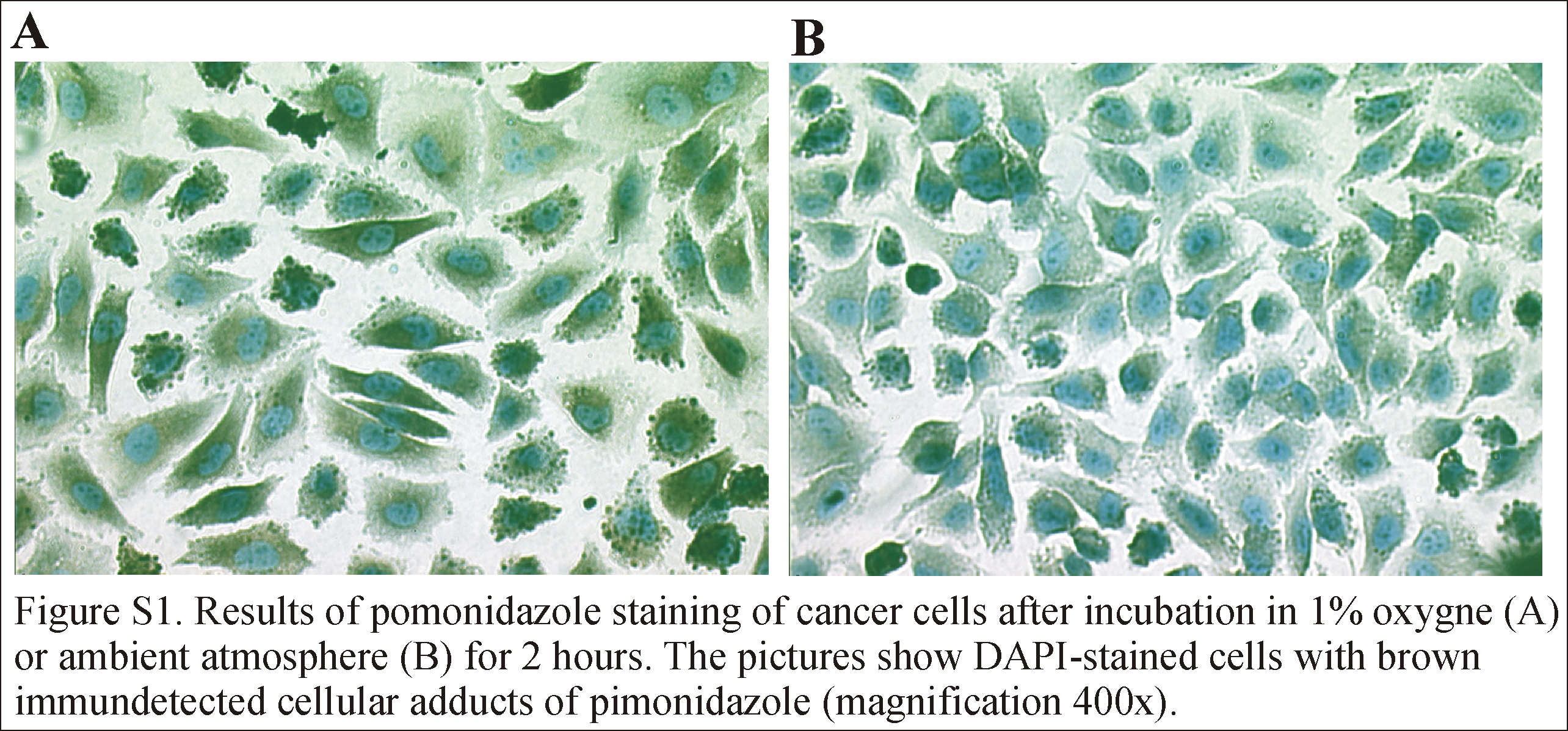

Supplement: Figure S1 — Pimonidazole-staining of WM793B cells after 2 hour incubation in hypoxic atmosphere (1% O2). (TIF) [file pone.0105104.s001.tif]
