# Supplementary material for: Global Gene Expression Profiling in Three Tumor Cell Lines Subjected to Experimental Cycling and Chronic Hypoxia
Source: PLoS One. 2014 Aug 14;9(8):e105104. doi: 10.1371/journal.pone.0105104 (PMC4133353; doi:10.1371/journal.pone.0105104)
Supplement: Table S1 — Sequence of the primers used for qRT-PCR analysis. (DOC) [file pone.0105104.s002.doc]

Table S1. Sequence of the primers used in qRT-PCR.

| **Gene** | **Forward primer** | **Reverse primer** | **Probe (Roche)** |
| --- | --- | --- | --- |
|  | **Reference genes** |  |  |
| *HADHA*  (NM_000182.4) | gtcttgcgcccatgatgt | cagcttcttcgggtcaactc | #65 |
| *EIF5*  (NM_001969.4) | tgggtttatgtctttatttgacga | gctatgtttccccaatacaggt | #46 |
| *CTBP1*  (NM_001328.2) | cgagtcggaacccttcag | cagatgaggttgggtgcat | #81 |
|  | **Experimental genes** |  |  |
|  | agacagcagagcacacaagc | cacagtgagatggttccttcc | #72 |
| *AREG*  *(*NM_001657.2) | ttgacagtagtttatcaaaaattgcat | cctgacgtattgtcttctaagctg | #46 |
| *HBEGF*  (NM_001945.2) | gtggggcttctcatgtttagg | gtcatgcccaacttcactttctc | #55 |
| *EPHA2*  *(*NM_004431.2) | ccaggcaggctacgagaa | ggctctcagatgcctcaaac | #88 |
| *PLAU*  (NM_001145031.1) | ttgctcaccacaacgacatt | ggcaggcagatggtctgtat | #46 |
| *CXCL2*  (NM_002089.3) | gaaaatcatcgaaaagatgctg | tgcagctgtgtctctctttcc | #83 |
