# Supplementary material for: Global Gene Expression Profiling in Three Tumor Cell Lines Subjected to Experimental Cycling and Chronic Hypoxia
Source: PLoS One. 2014 Aug 14;9(8):e105104. doi: 10.1371/journal.pone.0105104 (PMC4133353; doi:10.1371/journal.pone.0105104)
Supplement: Table S2 — Multivariate analysis. (DOC) [file pone.0105104.s003.doc]

**Table S2. Multivariate analyses of the treatment (hypoxia) effect in the context of cell line effect**

|  | **Hypoxia-related effect**  **(no of probe sets)** | **Cell line-related effect**  **(no of probe sets)** |
| --- | --- | --- |
| **Chronic hypoxia**  **vs. controls** | **8635** | **19011** |
| **Cycling hypoxia**  **vs. controls** | **6132** | **19316** |
| **Chronic hypoxia**  **vs. cycling hypoxia** | **4993** | **19295** |

Multivariate analysis of variance (MANOVA, FDR adjusted p<0.05) has been carried out three times, each time the treatment (column 1) and cell line effect were considered as two independent factors
