# Supplementary material for: Global Gene Expression Profiling in Three Tumor Cell Lines Subjected to Experimental Cycling and Chronic Hypoxia
Source: PLoS One. 2014 Aug 14;9(8):e105104. doi: 10.1371/journal.pone.0105104 (PMC4133353; doi:10.1371/journal.pone.0105104)
Supplement: Table S9 — Selected hypoxia-regulated genes common to all three tumor cell lines. (DOC) [file pone.0105104.s010.doc]

**Table 2. Hypoxia-regulated genes common to all three tumor cell lines**

**(FC>5 chronic hypoxia vs. control, FDR adjusted p<0.05)**

| **Gene** | **ID** | **SK-OV-3**  **Fold change** | **PC-3**  **Fold change** | **WM793B**  **Fold change** |
| --- | --- | --- | --- | --- |
| *ADM* | [202912_at](https://www.affymetrix.com/LinkServlet?probeset=202912_at) | 17.5 | 20.1 | 4.9 |
| *ALDOC* | [202022_at](https://www.affymetrix.com/LinkServlet?probeset=202022_at) | 12.8 | 5.3 | 21.8 |
| *ANG* | [205141_at](https://www.affymetrix.com/LinkServlet?probeset=205141_at) | 11.4 | 2.7 | 5.4 |
| [*ANGPTL4*](http://www.ncbi.nlm.nih.gov/entrez/query.fcgi?cmd=search&db=gene&term=ANGPTL4) | [223333_s_at](https://www.affymetrix.com/LinkServlet?probeset=223333_s_at) | 42.4 | 93.7 | 11.4 |
| *ANGPTL4* | [221009_s_at](https://www.affymetrix.com/LinkServlet?probeset=221009_s_at) | 67.8 | 70.2 | 15.3 |
| [*ANKRD37*](http://www.ncbi.nlm.nih.gov/entrez/query.fcgi?cmd=search&db=gene&term=ANKRD37)*^#^* | 227337_at | 23.8 | 11.0 | 19.3 |
| [***ARRDC3***](http://www.ncbi.nlm.nih.gov/entrez/query.fcgi?cmd=search&db=gene&term=ARRDC3) | [224797_at](https://www.affymetrix.com/LinkServlet?probeset=224797_at) | 6.6 | 7.1 | 5.0 |
| *BNIP3* | [201849_at](https://www.affymetrix.com/LinkServlet?probeset=201849_at) | 6.4 | 2.9 | 7.5 |
| *BNIP3* | [201848_s_at](https://www.affymetrix.com/LinkServlet?probeset=201848_s_at) | 6.7 | 4.4 | 12.9 |
| *CA9* | [205199_at](https://www.affymetrix.com/LinkServlet?probeset=205199_at) | 46.3 | 2.6 | 28.4 |
| *DDIT3* | 209383_at | 0.18 | 7.5 | 11.4 |
| [*ENO2*](http://www.ncbi.nlm.nih.gov/entrez/query.fcgi?cmd=search&db=gene&term=ENO2) | [201313_at](https://www.affymetrix.com/LinkServlet?probeset=201313_at) | 21.8 | 7.1 | 26.9 |
| [*HIG2*](http://www.ncbi.nlm.nih.gov/entrez/query.fcgi?cmd=search&db=gene&term=C7orf68) | [1554452_a_at](https://www.affymetrix.com/LinkServlet?probeset=1554452_a_at) | 8.9 | 10.9 | 7.1 |
| [*HIG2*](http://www.ncbi.nlm.nih.gov/entrez/query.fcgi?cmd=search&db=gene&term=C7orf68) | [218507_at](https://www.affymetrix.com/LinkServlet?probeset=218507_at) | 11.1 | 11.0 | 7.5 |
| [*INSIG2*](http://www.ncbi.nlm.nih.gov/entrez/query.fcgi?cmd=search&db=gene&term=INSIG2) | [209566_at](https://www.affymetrix.com/LinkServlet?probeset=209566_at) | 6.9 | 3.8 | 5.2 |
| *KLF7^#^* | [204334_at](https://www.affymetrix.com/LinkServlet?probeset=204334_at) | 5.7 | 13.8 | 2.6 |
| *LOXL2* | 202997_s_at | 14.5 | 5.9 | 2.2 |
| *MT1X^#^* | 204326_x_at | 5.1 | 5.3 | 0.4 |
| *NDRG1* | [200632_s_at](https://www.affymetrix.com/LinkServlet?probeset=200632_s_at) | 14.4 | 8.2 | 2.7 |
| [*PDK1*](http://www.ncbi.nlm.nih.gov/entrez/query.fcgi?cmd=search&db=gene&term=PDK1) | [226452_at](https://www.affymetrix.com/LinkServlet?probeset=226452_at) | 5.9 | 4.2 | 8.6 |
| *PFKFB4* | [228499_at](https://www.affymetrix.com/LinkServlet?probeset=228499_at) | 18.5 | 5.2 | 10.1 |
| *PPFIA4****^#^*** | [214978_s_at](https://www.affymetrix.com/LinkServlet?probeset=214978_s_at) | 7.7 | 3.7 | 9.3 |
| *RNASE4* | [205158_at](https://www.affymetrix.com/LinkServlet?probeset=205158_at) | 28.8 | 3.1 | 9.8 |
| [*SLC2A1*](http://www.ncbi.nlm.nih.gov/entrez/query.fcgi?cmd=search&db=gene&term=SLC2A1) | [201250_s_at](https://www.affymetrix.com/LinkServlet?probeset=201250_s_at) | 6.4 | 5.5 | 4.9 |
| *STC1^#^* | [204597_x_at](https://www.affymetrix.com/LinkServlet?probeset=204597_x_at) | 34.1 | 3.4 | 7.7 |
| *STC1^#^* | [204596_s_at](https://www.affymetrix.com/LinkServlet?probeset=204596_s_at) | 41.4 | 3.8 | 24.0 |
| *STC2^#^* | [203439_s_at](https://www.affymetrix.com/LinkServlet?probeset=203439_s_at) | 2.5 | 9.9 | 6.1 |
| [***TAF9B***](http://www.ncbi.nlm.nih.gov/entrez/query.fcgi?cmd=search&db=gene&term=TAF9B) | [228483_s_at](https://www.affymetrix.com/LinkServlet?probeset=228483_s_at) | 11.2 | 8.8 | 6.3 |
| *TMEM45A^#^* | [219410_at](https://www.affymetrix.com/LinkServlet?probeset=219410_at) | 11.3 | 3.1 | 5.5 |
| *VEGF* | [211527_x_at](https://www.affymetrix.com/LinkServlet?probeset=211527_x_at) | 3.7 | 12.7 | 9.5 |
| *VEGF* | [210513_s_at](https://www.affymetrix.com/LinkServlet?probeset=210513_s_at) | 2.8 | 11.4 | 7.7 |
| *VEGF* | [212171_x_at](https://www.affymetrix.com/LinkServlet?probeset=212171_x_at) | 3.4 | 6.5 | 5.6 |
| *ZNF395^#^* | [223216_x_at](https://www.affymetrix.com/LinkServlet?probeset=223216_x_at) | 9.7 | 3.4 | 5.2 |

In **bold** are new hypoxia-responsive genes, those marked with # have been

modestly reported to be associated with hypoxia so far.
